# Management of Ventilator-Associated Pneumonia Caused by *Pseudomonas* and *Acinetobacter* Organisms in a Pediatric Center: A Randomized Controlled Study

**DOI:** 10.3390/medicina60122098

**Published:** 2024-12-21

**Authors:** Mona Moheyeldin AbdelHalim, Seham Awad El Sherbini, El Shimaa Salah Ahmed, Heba Abdullah Abdalbaset Gharib, Marwa O. Elgendy, Ahmed R. N. Ibrahim, Heba Sherif Abdel Aziz

**Affiliations:** 1 Department of Clinical and Chemical Pathology, Faculty of Medicine, Cairo University, Cairo 12613, Egypt; mona.mohieedden@kasralainy.edu.eg; 2 Department of Pediatrics, Faculty of Medicine, Cairo University, Cairo12613, Egypt; awadseham@yahoo.com (S.A.E.S.); elshimaasalah@yahoo.com (E.S.S.A.); 3 Department of Pediatrics, Egyptian Ministry of Health, Cairo 71529, Egypt; hobagharib1788@gmail.com; 4 Department of Clinical Pharmacy, Beni-Suef University Hospitals, Faculty of Medicine, Beni-Suef University, Beni Suef 62521, Egypt; 5 Department of Clinical Pharmacy, Faculty of Pharmacy, Nahda University (NUB), Beni Suef 62764, Egypt; 6 Department of Clinical Pharmacy, College of Pharmacy, King Khalid University, Abha 61421, Saudi Arabia; aribrahim@kku.edu.sa

**Keywords:** ventilator-associated pneumonia, Ameri–Ziaei double antibiotic synergism test, multidrug resistance, extensively drug resistance, pandrug resistance

## Abstract

A dangerous infection contracted in hospitals, ventilator-associated pneumonia is frequently caused by bacteria that are resistant to several drugs. It is one of the main reasons why patients in intensive care units become ill or die. This research aimed to determine the most effective empirical therapy of antibiotics for better ventilator-associated pneumonia control and to improve patient outcomes by using the minimal inhibitory concentration method and the Ameri–Ziaei double antibiotic synergism test and by observing the clinical responses to both single and combination therapies. Patients between the ages of one month and twelve who had been diagnosed with ventilator-associated pneumonia and had been on mechanical ventilation for more than 48 h were included in the study, which was carried out in the Pediatric Intensive Care Unit at Cairo University’s Hospital. When ventilator-associated pneumonia is suspected, it is critical to start appropriate antibiotic therapy as soon as possible. This is especially important in cases where multidrug-resistant Gram-negative infections may develop. Although using Polymyxins alone or in combination is effective, it is important to closely monitor their administration to prevent resistance from increasing. The combination therapy that showed the greatest improvement was a mix of aminoglycosides, quinolones, and β-lactams. A combination of aminoglycosides and dual β-lactams came next. Although the optimal duration of antibiotic treatment for ventilator-associated pneumonia is still unknown, treatments longer than seven days are usually required to eradicate MDR *P. aeruginosa* or *A. baumannii* completely.

## 1. Introduction

Ventilator-associated pneumonia (VAP) is a severe infection acquired in hospitals, which is often linked to bacteria resistance to multiple drugs [1]. It is a leading cause of illness and death among patients in intensive care units (ICUs) [2]. Among pediatric patients, it is the most commonly identified nosocomial infection associated with medical devices, affecting 90% of cases [3]. Incidence rates of VAP in Egyptian University Hospitals range from 16 to 75% [4]. VAP typically develops at least two days after initiation of mechanical ventilation [5,6]. Regarding the onset of VAP, it may present as early or late onset, with late-onset cases being more common after four or more days of ventilation [7]. Risk factors associated with VAP include prolonged mechanical ventilation, immunodeficiency, steroid therapy, reintubation, and primary bloodstream infection [8].

Bacterial infections that are frequently multidrug-resistant are the leading cause of ventilator-associated pneumonia (VAP), with *Pseudomonas aeruginosa*, *Klebsiella* species, methicillin-resistant *Staphylococcus aureus* (MRSA), and *Acinetobacter* species being the most commonly involved pathogens [9]. Diagnosing VAP usually involves a combination of clinical evaluations, radiographic imaging, and microbiological tests. Important diagnostic indicators include the presence of new or worsening lung infiltrates alongside fever, alterations in white blood cell counts (either leukocytosis or leukopenia), purulent secretions, and deteriorating gas exchange [10,11]. With multidrug-resistant bacterial infections becoming more prevalent globally, new techniques for testing bacterial susceptibility to antibiotics are crucial for early and effective treatment [12]. Methods such as the Kirby–Bauer disk diffusion test (DD), Ameri–Ziaei double antibiotic synergism test (AZDAST), and minimal inhibitory concentration (MIC) are used for antimicrobial susceptibility testing (AST).

*Pseudomonas aeruginosa* (*P. aeruginosa*) is a Gram-negative opportunistic pathogen that primarily infects individuals with cystic fibrosis, burn wounds, compromised immune systems, chronic obstructive pulmonary disease (COPD), cancer, and severe infections requiring ventilation. This bacterium has the ability to adapt to harsh environments within its hosts by releasing various virulence factors that aid in infection and disease progression. One key factor is the lipopolysaccharide (LPS), an essential component of the bacterial surface that not only protects its outer membrane but also harms host cells. The endotoxic properties of lipid A within the LPS contribute to tissue damage and play a role in the bacterium’s attachment to and recognition by host receptors. Additionally, the LPS may be linked to antibiotic tolerance and biofilm development [12].

Outer membrane proteins (OMPs) facilitate nutrient exchange, adhesion, and antibiotic resistance. Furthermore, the development of drug resistance often attributed to biofilm formation is connected to structures like flagella, pili, and other adhesion factors. There are also six types of secretion systems, including those involving flagella (associated with T6SS), pili (T4SS), and multi-toxin components from the type 3 secretion system (T3SS). These systems are crucial for adhering to, and colonizing the host, and for responding to chemical signals. Additionally, exopolysaccharides such as alginate, Psl, and Pel contribute to biofilm formation and hinder bacterial clearance [8].

*Acinetobacter baumannii* is another Gram-negative bacteria considered an ESKAPE pathogen that poses significant public health risks due to its ability to cause severe invasive infections, mostly acquired in healthcare settings, which often result in high mortality rates. In recent years, this pathogen has shown multidrug resistance (MDR), which is largely due to excessive antibiotic use and inadequate management. MDR strains are often linked to prolonged hospital stays, the presence of catheters, and mechanical ventilation, with immunocompromised and severely ill patients being more susceptible to these invasive infections [4].

Advancements in next-generation sequencing have transformed the diagnosis of serious *A. baumannii* infections, allowing for quicker identification and tailored treatment plans based on the specific resistance genes involved. The excessive use of antibiotics, combined with the slow pace of developing new effective antimicrobials, poses major challenges. This situation emphasizes the need for innovative theoretical and practical approaches to screen and create novel drugs tested for their mechanisms to effectively tackle difficult infections, particularly those caused by multidrug-resistant strains [7].

Preventive measures, including hand hygiene [13], oral care, clean suctioning, stress ulcer prophylaxis, sedation vacation, and ventilator circuit changes, can help improve VAP outcomes [14]. Once VAP is suspected, empirical antibiotic therapy should be initiated based on local hospital antibiograms. Combination therapy from different antibiotic classes is necessary for patients at risk of infection with multidrug-resistant Gram-negative bacteria (MDR-GNB). Subsequently, antibiotics should be tailored based on culture and antibiotic susceptibility test results of the causative pathogen [15]. Antimicrobial treatment should ideally not exceed 7 days, although longer durations may be necessary for treating pneumonia caused by *Pseudomonas* or *Acinetobacter* species or in cases of complications [2].

All antibiotics that have effectively treated bacterial infections when used as standalone treatments have relied on complex mechanisms to inhibit bacterial growth rather than just blocking a single enzyme. These effective antibiotics typically work by targeting multiple sites simultaneously or they involve targets that are controlled by several genes. This diversity in targets and genes helps slow down the development of resistance related to these targets. Recent approvals of antibiotics and new ones in the development pipeline are being examined in the context of this encouraging idea of using multiple drugs to combat bacterial infections.

This study aimed to determine the most effective empirical therapy of antibiotics for managing ventilator-associated pneumonia and enhancing patient outcomes. It utilized the minimal inhibitory concentration method and the Ameri–Ziaei double antibiotic synergism test while assessing clinical responses to both monotherapy and combination therapy approaches.

## 2. Materials and Methods

### 2.1. Settings and Study Design

**Figure 1:**
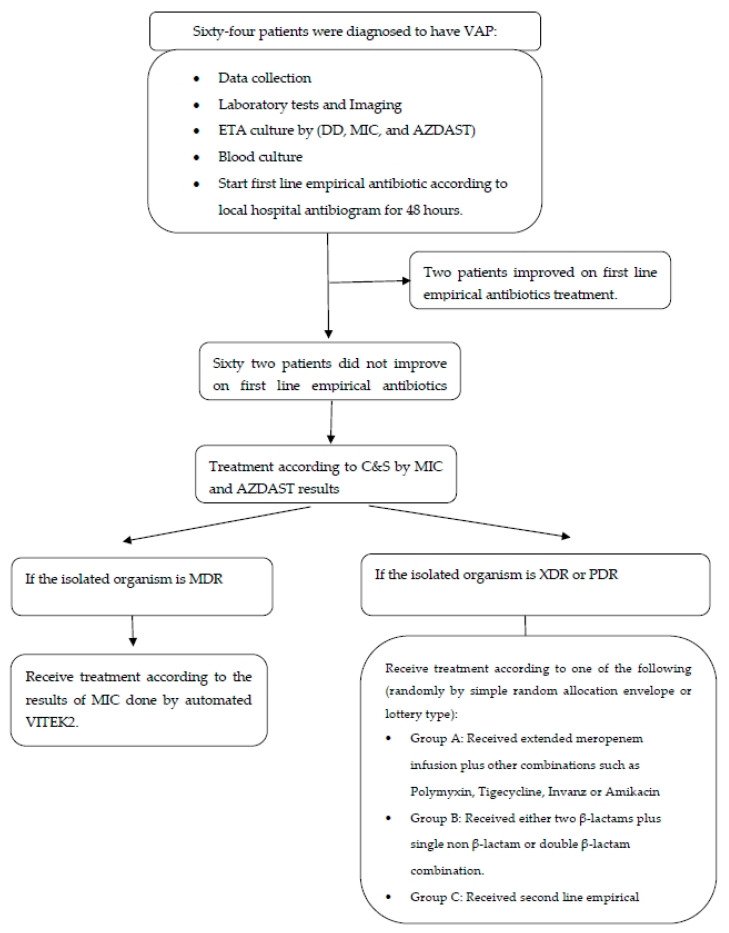
Flowchart of the work.

This prospective randomized controlled intervention study took place in the Pediatric Intensive Care Unit (PICU) at Cairo University’s Faculty of Medicine from January 2021 to January 2022. Sixty-four critically ill patients diagnosed with VAP were included in the study. Figure 1 show the flow chart for this study.

#### 2.1.1. Ethical Approval

Approval for the study was granted by the Research Ethical Committee of Cairo University’s Faculty of Medicine (code MS-280-2020). Informed consent for participation and publication was obtained from the legal guardians of the patients.

#### 2.1.2. Inclusion Criteria

Patients ranging from one month to twelve years old, who had been undergoing mechanical ventilation for over 48 h and were diagnosed with VAP, were included in the study. The clinical criteria for diagnosing VAP were established by the National Nosocomial Infections Surveillance (NNIS) and the Centers for Disease Control and Prevention (CDC) [16].

#### 2.1.3. Exclusion Criteria

Patients with pneumonia prior to mechanical ventilation initiation were not included in the study. Additionally, patients who had been intubated for more than 24 h before admission to the PICU, or those transferred from other PICUs and were already receiving antibiotic therapy, were also excluded from the study.

### 2.2. Data Collection

#### 2.2.1. Each Patient Underwent the Following Assessments

Upon admission, the following data were gathered: demographic information such as age, sex, and place of residence; the primary reason for admission to the PICU, categorized as respiratory, cardiac, circulatory, neurological, or other causes; an assessment of systemic failure and grading of sepsis [17]; the Pediatric Logistic Organ Dysfunction (PELOD)-2 Score [18]; the Pediatric Risk of Mortality (PRISM) III score [19]; and details of received treatments, including empirical antibiotics.

Throughout their hospital stay, the following data were collected: daily vital signs, such as blood pressure, heart rate, respiratory rate, and oxygen saturation; comprehensive systemic examinations; duration of mechanical ventilation and ventilator settings; oxygen saturation index; sepsis grading and signs of multiple organ system failure (MOSF); length of hospital stay; and clinical outcomes (discharge or death).

#### 2.2.2. Imaging and Laboratory Investigations

Upon suspicion of VAP, the following tests were conducted: complete blood count (CBC), C-reactive protein (CRP), cultures of endotracheal tube aspirates (ETA) and blood, liver function tests, renal function tests, arterial blood gases (ABG), and chest X-rays.

Laboratory examinations were repeated 48 h after initiation of empirical antibiotic(s), which was followed by weekly monitoring of laboratory tests and chest X-rays to track patient progress.

### 2.3. Diagnostic Intervention

Upon diagnosis of VAP, an endotracheal aspirate was obtained for culture and sensitivity testing. Antimicrobial susceptibility was assessed through the Kirby–Bauer disk diffusion method (DD), minimal inhibitory concentration (MIC) using the automated VITEK 2 compact system (bioMérieux, Craponne, France), and the Ameri–Ziaei double antibiotic synergism test (AZDAST). Interpretation of DD and MIC results followed CLSI guidelines [20].

The Ameri–Ziaei double antibiotic synergism test (AZDAST) was performed to test the isolated bacteria against four combinations (each combination included triple antibiotics). These combinations were:

A—Ciprofloxacin + Cefoxitin + Cefepime
B—Ceftazidime/avibactam + Meropenem + Gentamycin
C—Ciprofloxacin+ Piperacillin/tazobactam + Amikacin
D—Piperacillin/tazobactam + Amikacin + Cefoxitin

Subsequently, results were interpreted according to Ziaei-Darounkalaei et al. [21].

### 2.4. Therapeutic Intervention

Upon diagnosing VAP, we initiated an empirical treatment regimen based on the hospital’s annual antibiogram. This regimen consisted of a combination of beta-lactam antibiotics such as Piperacillin/tazobactam, imipenem, Meropenem, Cefepime, or Ceftazidime, along with non-beta-lactam antibiotics including Ciprofloxacin, Levofloxacin, Amikacin, Gentamycin, or Polymyxin. Subsequently, antibiotics were adjusted according to culture and sensitivity results obtained via MIC or the AZDAST, and were selected randomly through a simple random allocation envelope or lottery type as follows:

(1) If MDR *Pseudomonas* spp. or *Acinetobacter* spp. were isolated from the patient’s specimen, treatment was modified based on antibiotic susceptibility results obtained via MIC.
(2) In cases of isolation of XDR or PDR *Pseudomonas* spp. or *Acinetobacter* spp. from the patient’s specimen, treatment was adjusted as follows:
  (a) Group A patients: treatment was modified to include extended Meropenem infusion in combination with other options such as Polymyxin, Tigecycline, Ertapenem, or Amikacin [22].
  (b) Group B patients: treatment was adjusted to include two β-lactam antibiotics plus a single non-β-lactam antibiotic, or a combination of double β-lactam antibiotics, with the aim of avoiding aggressive and toxic antibiotics [23].
  (c) Group C patients: treatment was adjusted to include the second-line empirical antibiotic according to the hospital’s local policy.

### 2.5. Statistical Analysis

- The collected data were computerized and analyzed using the Statistical Package for Social Sciences (SPSS) version 28.
- Qualitative data were represented as frequencies and relative percentages.
- Quantitative data were expressed as mean ± SD (standard deviation) for normally distributed data and as median and range for non-normally distributed data.
- The Wilcoxon test was employed to compare pre- and post-treatment outcomes.
- A significance level of *p*-value ≤ 0.05 indicated significance; *p* < 0.001 indicated a highly significant difference, while *p* > 0.05 indicated a non-significant difference.

## 3. Results

### 3.1. Demographic Data of the Patients

In the current study, 64 patients were enrolled, where 56.3% of them were males and 43.7% were females, and the mean ± SD of age was 27.9 ± 31.5 months. In total, 45.3% of patients had neurological problems, which was the most common system affection, followed by chest infection (17.2%), gastrointestinal tract disorders (12.5%), MOSF (10.9%), cardiovascular diseases (9.4%) and renal affection (4.7%). The mean ± SD of the PRISM III score was 12 ± 7 and the mean ± SD of the PELOD-2 score was 8 ± 4. The mean ± SD of the duration of hospital stay/day was 52 ± 30 days.

Regarding the clinical outcome of VAP patients, 49 (76.6%) patients improved and 15 (23.4%) patients did not improve (deteriorated or died).

### 3.2. Distribution of Different Microorganisms Among Microbiological Cultures

ETA cultures revealed that the most common isolated organism was *Pseudomonas aureginosa* in 48.4% (n = 31) followed by *Acinetobacter* spp. in 31.3% (n = 20), combined *Pseudomonas aureginosa* and *Klebsiella* spp. in 7.8% (n = 5), combined *Acinetobacter* spp. and *Klebsiella* spp. in 6.3% (n = 4), combined *P. aureginosa* and *Acinetobacter* spp. in 3.1% (n = 2), and combined *P. aureginosa* and MRSA in 3.1% (n = 2).

Blood cultures revealed no growth in 68.8% of cases (n = 44), Coagulase-Negative *Staphylococcus* in 21.9% (n = 14), *Klebsiella* spp. in 4.7% (n = 3), *Candida* spp. in 1.6% (n = 1.6), and MRSA in 3.1% (n = 2).

### 3.3. Antibiotic Resistance Pattern of the Isolated Microorganisms

Multidrug-resistant microorganisms were isolated from seven patients and pandrug-resistant microorganisms were isolated from ten patients, while extensive drug-resistant microorganisms were isolated from forty-seven patients. The distribution of MDR, PDR, and XDR microorganisms among patients who clinically improved versus clinically did not improve is illustrated in Table 1.

#### 3.3.1. Analysis of AZDAST Results

Table 2 illustrates the difference between the four tested AZDAST combinations regarding synergism, potentiation, and antagonism. Combination A has more antagonism than potentiation (*p* value= 0.007), while combination B has more potentiation than antagonism (*p* value= 0.018).

#### 3.3.2. Analysis of Antibiotic Use

Table 3 shows the frequency of empirically used antibiotics according to the local hospital antibiogram; the most commonly used antibiotic was beta-lactam (68,6%), followed by double beta-lactam (17.2%), a combination of beta-lactam and aminoglycoside (9.4%), quinolone (3.1%), and aminoglycoside (1.6%).

Table 4 shows 37 patients received antibiotics according to the MIC results, where the most frequent antibiotic used was Polymyxin (94.6%), followed by beta-lactam and aminoglycoside (2.7%), and aminoglycoside and Polymyxin (2.7%).

Table 5 shows that 15 patients received antibiotics according to the AZDAST results; 53.3% of them received aminoglycoside, quinolones, and beta-lactam, while 46.7% of them received aminoglycoside and double beta-lactam.

Table 6 shows that the antibiotic therapy of 22 patients was shifted empirically to the second-line antibiotics, according to the local hospital antibiogram, as follows: beta-lactam and quinolones (31.8%), Polymyxin and quinolones (18.2%), beta-lactam (18.2%), Polymyxin and beta-lactam (13.6%), Polymyxin, quinolones, and beta-lactam (9.1%), Polymyxin and double beta-lactam (4.5%), and aminoglycosides and beta-lactam (4.5%).

#### 3.3.3. Clinical Outcome Analysis

There is no statistically significant difference regarding clinical scores and laboratory studies in relation to VAP outcome, as shown in Table 7.

Most of the patients (96.9%) who received first-line empirical antibiotics did not improve and were shifted to another line of antibiotics; 45.2% of them were shifted to the Group A treatment, 19.4% were shifted to the Group B treatment, and 35.5% were shifted to the Group C treatment (Table 8).

**Figure 2:**
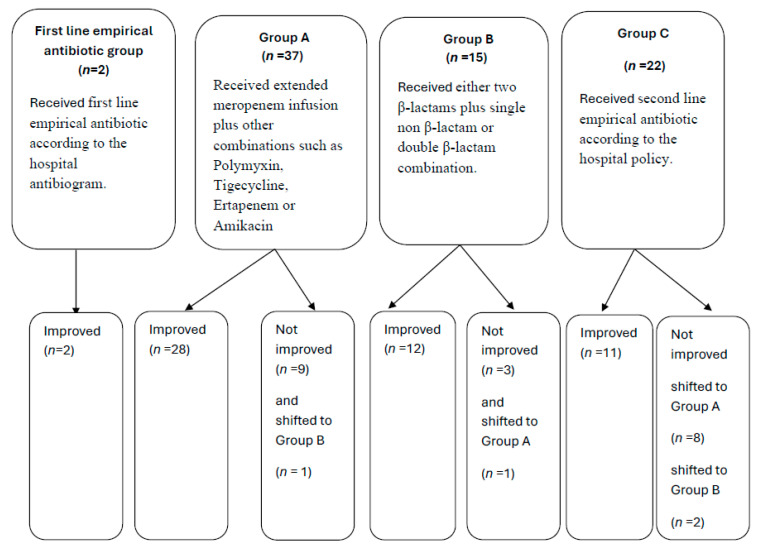
Summary of the clinical outcome of the studied population (n = 64).

Figure 2 shows a summary for the clinical outcome of the studied population.

Patients in Group C significantly showed a need to shift to another antibiotic regimen compared to patients in Groups A and B (*p*-value < 0.0011, 0.011) (Table 9).

## 4. Discussion

VAP is a frequent complication in patients receiving ventilatory support for acute respiratory failure [24,25], posing significant clinical and economic challenges in critically ill individuals [26]. Consequently, the prompt initiation of appropriate empirical antimicrobial therapy is essential upon suspicion of VAP, taking into account factors such as prior antibiotic exposure, patient comorbidities, the duration of hospital stay, local epidemiology, and the hospital antibiogram [27]. MDR organisms such as *P. aeruginosa*, *Acinetobacter baumanii*, methicillin-resistant *Staphylococcus aureus* (MRSA), extended-spectrum beta-lactamase-producing Enterobacteriaceae (ESBL-E), and carbapenemase-producing Enterobacteriaceae (CPE) are commonly implicated in VAP [28].

In our research, we concentrated on VAP episodes triggered by MDR *P. aeruginosa* and *Acinetobacter* species with the goal of identifying the most effective empirical antibiotic therapy to improve VAP management and outcomes, employing AZDAST and MIC methods, and assessing the clinical response to both monotherapy and combination therapy. Our study comprised 64 VAP-diagnosed patients, with 56.3% being male and 43.8% female and an average age of 27.9 ± 31.5 months. Neurological diseases were the most prevalent clinical diagnoses associated with VAP in our study, which is consistent with the findings from Malhotra, P et al. (2018) [29] and Galal et al. (2016) [30], possibly due to prolonged mechanical ventilation resulting from multiple risk factors such as impaired consciousness, muscle weakness, and diminished cough reflex in these cases. In contrast, a study by Chiru et al. (2013) reported that acute respiratory failure followed by severe sepsis were the most common clinical diagnoses in VAP patients aged 0–18 years [30]. We observed leukocytosis and elevated C-reactive protein levels in all patients, which is consistent with the findings of Ghattas et al. (2022) [31], while Manjhi et al. (2018) reported that fever, leukocytosis, and leucopenia were not significant predictors of VAP [32]. Additionally, we noted that blood cultures had limited value in predicting VAP severity and demonstrated low sensitivity in detecting the same organism isolated from respiratory specimen cultures, which is consistent with previous studies [31,33]. However, other studies have suggested performing blood cultures for all VAP-diagnosed patients as they may help identify the responsible pathogen, especially when respiratory cultures are inconclusive, which is in line with research by Kalil et al. (2016) and Modi and Kovacs (2020) [2,34].

Our research revealed that MDR *Pseudomonas aureginosa* was the predominant organism responsible for VAP, accounting for 48.4% of cases, compared to MDR *Acinetobacter* spp. accounting for 31.3%, which aligns with the findings from studies conducted by Galal et al. (2016) and Bhattacharya et al. (2023) [30,35]. Conversely, Mahantesh et al. (2017) found that *Acinetobacter* spp. were the most commonly isolated bacteria in VAP cases [36]. We observed that combined infections involving multiple causative organisms, such as either MDR *Pseudomonas* species and *Acinetobacter* species or one of them along with *Klebsiella* species or MRSA, occurred in 20.3% of our study group, which is consistent with the findings of Mahantesh et al. (2017) [36]. However, it was less common to encounter more than one organism, as noted by Vedavathy et al. (2016) [37].

In terms of antibiotic resistance, our study found that 10.9% of isolates were MDR, 73.4% were XDR, and 15.6% were PDR based on the antibiotics tested in our microbiology laboratory, which corresponds closely to the findings of Minh et al. (2022) [38]. Our MIC results showed high sensitivity to Polymyxin (94.6%), followed by β-lactam and aminoglycoside (2.7%), and aminoglycoside combined with Polymyxin (2.7%). This is consistent with the findings of Tunyapanit et al. (2019) [39], who concluded that colistin remained the most effective antimicrobial agent against imipenem-resistant *Pseudomonas aeruginosa* isolates. Additionally, a study by Olsson et al. (2020) [40] indicated that Polymyxin combinations with Aztreonam, Cefepime, or Meropenem were highly active against MDR *Pseudomonas aeruginosa*, likely due to the membrane-disrupting properties of Polymyxins, facilitating the entry of a second antibiotic and counteracting decreased permeability and increased efflux. However, Ergul et al. (2017) [41] reported toxic side effects associated with colistin and its limited effectiveness in treating pulmonary infections, a sentiment echoed by Cisneros et al. (2019) [42].

In our investigation, we assessed the sensitivity of multidrug-resistant *Pseudomonas aeruginosa* and *Acinetobacter* strains to four antibiotic combinations using the AZDAST. We observed that the combination of quinolone and double β-lactams (Ciprofloxacin, Cefepime, and Cefoxitin) exhibited more antagonism than potentiation. Conversely, the combination of aminoglycoside and double β-lactams (Gentamicin, Ceftazidime/avibactam, and Meropenem) showed more potentiation than antagonism. Furthermore, we noted a high percentage of synergism and potentiation with combinations such as aminoglycoside, quinolone, and β-lactam (Amikacin, Ciprofloxacin, and Piperacillin/tazobactam), and aminoglycoside combined with double β-lactams (Gentamicin, Ceftazidime/avibactam, and Meropenem or Amikacin, Cefoxitin, and Piperacillin/tazobactam).

These findings are consistent with Sun et al. (2011) [43], who reported the highest likelihood of synergy with an aminoglycoside combined with an antipseudomonal penicillin, followed by a cephalosporin or a carbapenem. Additionally, Yasmin et al. (2013) found synergistic or additive effects with Amikacin plus Ciprofloxacin against *Pseudomonas aeruginosa* [44]. However, Tunyapanit et al. observed no synergy with Amikacin plus Ciprofloxacin in all of the IRPA and high-level IRPA isolates tested [39]. They also found antagonism in some isolates. This may be explained by resistance to fluoroquinolones, which depends on the action of efflux pumps which are either intrinsic, such as the resistance-nodulation-division (RND) family of efflux pumps, or are acquired through mutations to the genes regulating the production of efflux pumps, leading to the overexpression of efflux pumps such as the MexAB-OprM [45]. Resistance of A. baumannii to fluoroquinolones is mediated through mutations in the genes of DNA gyrase and topoisomerase IV, reducing their affinity for fluoroquinolones, and through the production of qnr-type protection proteins, which inhibit the binding of fluoroquinolones to topoisomerase IV and DNA gyrase [46].

Contrary to these findings, a previous study demonstrated that empirical treatment with non-optimized double β-lactams (DβL) achieved similar clinical and microbiological responses and significantly better safety compared to β-lactam plus aminoglycoside (βLAG) therapy [47].

Our recent investigation revealed that only two patients showed improvement when treated with single empirical antibiotics. The vast majority (96.9%) of patients who received first-line empirical antibiotics did not show improvement and, consequently, required a shift to another line of antibiotics. Among these, 45.2% were switched to MIC, 19.4% to the AZADAST, and 35.5% to a second empirical antibiotic. Notably, patients treated with MIC showed significantly better improvement compared to those who received a second empirical antibiotic (*p*-value = 0.044).

In our study, approximately 80% of multidrug-resistant *Pseudomonas aeruginosa* and *Acinetobacter* strains showed sensitivity to Colistin, which was associated with greater improvement in VAP and a reduced need for switching to alternative antibiotics. Previous research has indicated that the addition of Polymyxin can enhance the intracellular concentrations of minocycline, leading to synergistic effects against *Acinetobacter baumannii* [40,48]. However, it is worth noting that Yilmaz et al. reported a 13% resistance rate to colistin among *Pseudomonas aeruginosa* isolates [49].

Furthermore, our findings indicated that all patients treated with Amikacin, Ceftriaxone, and Meropenem as single antibiotics did not experience improvement and required a change in antibiotic regimen. This aligns with the study by Ergul et al., which highlighted widespread carbapenem resistance among Gram-negative bacteria [41]. Studies by Ruppé et al. and Ning et al. demonstrated varying degrees of resistance among *Pseudomonas aeruginosa* and *Acinetobacter baumannii* strains to different antibiotic classes. Notably, *Acinetobacter baumannii* exhibited resistance to aminoglycosides, quinolones, cefotaxime, Piperacillin/tazobactam, and cefoperazone/sulbactam, making imipenem and Meropenem unfavorable choices for treating infections caused by this pathogen [50,51].

The resistance rates of *Pseudomonas aeruginosa* to imipenem and Meropenem were reported as 0% and 72.73%, respectively, by Ning et al. (2013) [52]. Conversely, Xu et al. (2016) found that only 4.2% of *Pseudomonas* strains were resistant to carbapenems [53]. The limited efficacy of Amikacin may be attributed to efflux pumps and genes encoding aminoglycoside-modifying enzymes, which deactivate most aminoglycosides except Plazomicin, a new aminoglycoside capable of overcoming their effect, as highlighted by Ramirez et al. (2017) and Behzadi et al. (2021) [45,54].

Resistance against β-lactams, as discussed by Kakoullis et al. (2021) and Behzadi et al. (2021), can arise due to various mechanisms, including influx pumps; β-lactamases such as AmpC β-lactamases and extended-spectrum β-lactamases (ESBLs) [46]; mutations in porins, such as a deficiency of the OprD porin leading to high-level resistance to imipenem and other carbapenems; overexpression of hydrolyzing enzymes like AmpC; overexpression of efflux pumps such as MexCD–OprJ reducing susceptibility to carbapenems; modification of PBPs reducing susceptibility to several β-lactams; and acquisition of other β-lactamases such as Class B carbapenemases [45,46].

In our study, patients were advised to undergo a prolonged course of antibiotic treatment for approximately 10 ± 2.5 days to effectively manage VAP. This aligns with the recommendation by Pugh et al. (2015), who suggested that pneumonia caused by *Pseudomonas* or *Acinetobacter* species should be treated for at least 2 weeks due to the risk of relapse associated with shorter durations [55]. Similarly, European guidelines recommend extended antibiotic therapy for XDR-GNB infections or bacteremia (Torres et al., 2017) [56].

In contrast, American and European guidelines suggest short-course antibiotic regimens (7 or 8 days) for VAP treatment, as supported by studies such as those by Elgendy, S.O et al. (2022) and Antalová et al. (2022) [15,51]. Prolonged antibiotic courses are discouraged as they can contribute to the emergence of resistance without providing significant benefits in terms of mortality, treatment failure, recurrent pneumonia, or duration of mechanical ventilation [15,51].

There is no statistically significant difference regarding clinical scores and laboratory studies in relation to VAP outcome. That may be due to VAP outcomes likely being influenced by a combination of factors beyond the measured variables, the adequacy of early treatment, immune responses, or other unmeasured clinical or environmental variables.

### Limitations

- Further multi-center studies across all regions of the country are needed to address the varying resistance rates in our hospitals.
- There were no previous antibiogram studies conducted in our hospitals for a comprehensive year-to-year comparison of resistance rates.

## 5. Conclusions

MDR *Pseudomonas aeruginosa* stands out as the predominant organism responsible for VAP. Prompt initiation of empirical antimicrobial therapy is crucial when VAP is suspected, particularly in cases where there is a risk of multidrug-resistant Gram-negative infections. While Polymyxin use, either alone or in combination with other regimens, demonstrates effectiveness, its usage must be regulated to mitigate the development of resistance. Among the various combinations, the most effective one that showed significant improvement comprises an aminoglycoside, quinolone, and β-lactam, followed by a combination of an aminoglycoside and double β-lactams. Though the definitive duration of antibiotic treatment for VAP remains undetermined, a regimen exceeding 7 days is typically necessary for the proper eradication of MDR *Pseudomonas aeruginosa* or *Acinetobacter baumannii.*

### Recommendations

Tailored clinical guidelines should be established for each healthcare facility, informed by local antibiogram data, to guide empirical treatment protocols. Further research involving larger study populations is needed to enhance the application of the AZDAST technique as a tool to test different antibiotic combinations.

Identifying risk factors associated with MDR organisms is crucial for better management. Implementation of appropriate antibiotic stewardship is essential to curb the escalating emergence of bacterial resistance. Employing proper in vitro susceptibility testing and expediting laboratory workup procedures to narrow down the antimicrobial spectrum with minimal turnaround time is imperative. Development of novel antimicrobial agents to serve as alternative therapeutic options against MDR Gram-negative pathogens is warranted. Prudent utilization of newly approved antibiotics is necessary to mitigate the risk of resistance. Careful evaluation of combined antimicrobial therapies is essential to mitigate potential adverse events.

## Figures and Tables

**Table 1:** The distribution of MDR, PDR, and XDR microorganisms among the studied population.

Number of Patients	Multidrug-Resistant Organisms	Pandrug-Resistant Organisms	Extensively Drug-Resistant Organisms
Total studied population n = 64 (100%)	7 (10.9%)	10 (15.6%)	47 (73.4%)
Patients who clinically did not improve n = 15	1 (14.3%)	3 (30.0%)	11 (23.4%)
Patients who clinically improved n =49	6 (85.7%)	7 (70%)	36 (76.6%)

**Table 2:** The results of the four tested AZDAST combinations.

	Total Number	Synergism	Potentiation	Antagonism	Additive	Not Distinguishable	*p*-Value Synergism and Potentiation Versus Antagonism
Combination A							
Ciprofloxacin + Cefoxitin + Cefepime	64	3 (4.7%)	5 (7.8%)	11 (17.2%)	0 (0%)	45 (70.3%)	0.007 *
Combination B							
Ceftazidime/avibactam + Meropenem + Gentamycin	64	13 (20.3%)	19 (29.7%)	6 (9.4%)	0 (0%)	26 (40.6%)	0.018 *
Combination C							
Ciprofloxacin + Piperacillin/tazobactam + Amikacin	64	15 (23.4%)	18 (28.1%)	13 (20.3%)	0 (0%)	18 (28.1%)	0.619
Combination D							
Piperacillin/tazobactam + Amikacin+ Cefoxitin	64	7 (10.9%)	22 (34.4%)	16 (25.0%)	1 (1.6%)	18 (28.1%)	0.437

* refers to significance.

**Table 3:** The first-line empirical antibiotics received once VAP was suspected.

Antibiotic Type	Frequency	Percentage
Aminoglycosides	1	1.6
Quinolones	2	3.1
Beta-lactam and aminoglycosides	6	9.4
Double beta-lactams	11	17.2
Beta-lactam	44	68.8
Total	64	100

**Table 4:** The antibiotics received according to the MIC results.

Antibiotic Type	Frequency	Percent
Aminoglycoside and Polymyxin	1	2.7
Beta-lactam and aminoglycosides	1	2.7
Polymyxin	35	94.6
Total	37	100

**Table 5:** The antibiotics received according to the AZDAST results.

Antibiotic Type	Frequency	Percentage
Aminoglycoside and double beta-lactam	7	46.7
Aminoglycoside, quinolone, and beta-lactam	8	53.3
Total	15	100

**Table 6:** The antibiotic received as second-line empirical therapy.

Antibiotic Type	Frequency	Percentage
Aminoglycoside and beta-lactam	1	4.5
Polymyxin and double beta-lactam	1	4.5
Polymyxin, quinolones, and beta-lactam	2	9.1
Polymyxin and beta-lactam	3	13.6
Beta-lactam	4	18.2
Polymyxin and quinolones	4	18.2
Beta-lactam and quinolones	7	31.8
Total	22	100

**Table 7:** The clinical scores and the laboratory parameters of the studied population.

	Clinical Outcome		The Test Result	*p*-Value
	Improved	Not Improved		
	n = 49	n = 15		
	Mean ± SD	Mean ± SD		
PRISM III score	12 ± 7	12 ± 8	−0.66	0.509
PELOD-2	8 ± 4	7 ± 5	−0.42	0.672
PIP	18 ± 3	20 ± 4	−1.77	0.078
PEEP	6 ± 1	6 ± 1	−0.90	0.371
Oxygen saturation index	6.6 ± 3.4	8.1 ± 4.7	−0.75	0.456
PH	7.35 ± 0.07	7.35 ± 0.06	−0.02	0.981
HCO3	28.1 ± 10.2	27.1 ± 7.8	−0.22	0.824
PaCo2	45 ± 14.4	45.7 ± 11.9	−0.03	0.975
BE	1.6 ± 5.4	1.1 ± 5.1	−0.22	0.824
HB	10.4 ± 1.8	10.1 ± 1.4	−0.16	0.874
PLT	291.7 ± 197.9	235.3 ± 151.2	−0.94	0.346
WBCs	16.2 ± 6.5	39.9 ± 84.8	−0.92	0.358
CRP	113 ± 59.6	120.1 ± 51.9	−0.39	0.698
Urea	20.8 ± 20.9	18.7 ± 14.7	−0.02	0.987
Creatinine	0.54 ± 0.87	0.42 ± 0.18	−0.24	0.809
ALT	69 ± 63	36 ± 17	−1.73	0.084
AST	79 ± 82	99 ± 138	−0.03	0.975

Data expressed as mean ± SD.

**Table 8:** Analysis of the clinical outcome of patients after receiving first-line therapy, the need to shift to another regimen, and the type of this regimen.

	Result	Frequency (Percentage)
Improvement after receiving first-line empirical regimen (n = 64)	No	62 (96.9%)
	Yes	2 (3.1%)
Shift to another regimen (n = 64)	No	2 (3.1%)
	Yes	62 (96.9%)
Type of regimen which the treatment was shifted to (n = 62)	Group A	28 (45.2%)
	Group B	12 (19.4%)
	Group C	22 (35.5%)

Patients in Group A significantly improved compared to patients in Group C (*p*-value = 0.044).

**Table 9:** Analysis of the clinical outcomes of the patients in the three groups of therapy.

		Group A n = 37	Group B n = 15	Group C n = 22	Group B Versus Group A	Group A Versus Group C	Group C Versus Group B
Improvement	No	9 (24.3%)	3 (20.0%)	11 (50%)	0.737	0.044 *	0.065
	Yes	28 (75.7%)	12 (80.0%)	11 (50%)			
Shift	No	36 (97.3%)	14 (93.3%)	12 (54.5%)	0.501	<0.001 **	0.011 *
	Yes	1 (2.7%)	1 (6.6%)	10 (45.4%)			
Shift to	Group A	0	1 (100%)	8 (80%)	---	---	---
	Group B	1 (100%)	0 (0%)	2 (20%)	---	---	---
	Group C	0(0%)	0(0%)	0(0%)	---	---	---

* refers to significance; ** refers to high significance.

## Data Availability

The datasets used and/or analyzed during the current study are available from the corresponding author on reasonable request.
